# Portuguese Version of the Oral Frailty Index-8: Instrument Validation Study

**DOI:** 10.2196/49975

**Published:** 2024-10-28

**Authors:** Laura Corrêa, André Júdice, Robson Scoz, Vanessa Machado, José João Mendes, Luís Proença, João Botelho, Luciano Ferreira

**Affiliations:** 1 Egas Moniz School of Health and Science Almada Portugal

**Keywords:** oral frailty, oral health, functional disability, frailty, aging, dentistry, confirmatory factor analysis, psychometric validity, questionnaire development

## Abstract

**Background:**

The concept of oral frailty has gained scientific and clinical relevance in recent years, and early detection can facilitate timely intervention to manage its progression. The Oral Frailty Index-8 (OFI-8) was developed to assess community-dwelling older adults at risk for oral frailty.

**Objective:**

This study aims to investigate the psychometric validity of the OFI-8 in the Portuguese population, named the Portuguese version of the OFI-8 (OFI-8-PT), which may serve as a reference for future studies related to longevity and oral function.

**Methods:**

This study included 2 main phases, involving patients aged 60 years or older, Portuguese speakers, and those who consented to participate in the study. First, the researchers translated and cross-culturally adapted the original questionnaire to make it suitable for native Portuguese speakers. The translated tool was then assessed for psychometric validation, which consisted of test-retest reliability, internal consistency, construct validity, and sex invariance measurement.

**Results:**

A total of 159 older adults participated in the baseline survey, with almost equal numbers of male (n=79, 49.7%) and female participants (n=80, 50.3%). The OFI-8-PT demonstrated good reliability (Cronbach α=0.95) and construct validity (goodness-of-fit index=0.96; comparative fit index=0.85; and root mean square error of approximation=0.05, 90% CI 0.00-0.09). The study found sex invariance, indicating that the OFI-8-PT is equally valid for male and female participants, and the tested-retest reliability of the OFI-8-PT was good, indicating consistent results over time.

**Conclusions:**

The OFI-8-PT showed psychometric validity and good reliability to be used in the Portuguese population.

## Introduction

Contemporary societies are grappling with an unprecedented aging phenomenon, propelled by the evolving age distribution of the global population [1,2], largely owing to remarkable gains in life expectancy [3]. The increasing longevity—considered a testament to medical advancements, improved access to treatments, and an emphasis on preventive care including oral health maintenance [1,4,5]—presents a notable societal achievement. However, amid this transition emerges the challenge of frailty—a condition marked by heightened vulnerability attributed to age-related declines in physiological reserves [6,7]. Frailty, often exacerbated by a prolonged lifespan, poses distinct health and societal complexities [2], underscoring the multifaceted nature of aging in contemporary times.

Within this spectrum, oral frailty was introduced in 2013 in Japan and has gained increased attention in recent years [8,9]. Oral frailty is defined as a decline in oral health and functional capacity, with broader implications for overall health and quality of life [8,9]. Tanaka et al [10] developed an 8-item questionnaire, referred to as the Oral Frailty Index-8 (OFI-8), for the purpose of surveying community-dwelling older adults who are at risk of oral frailty. Evidence suggests that early identification and intervention can slow the progression of oral frailty and may even prevent its onset [11,12]. This has led to a growing international interest in identifying the effects of aging on oral health and appropriate strategies for its prevention [11,13]. For this reason, oral frailty is currently considered one of the main determinants of oral health and dentistry policies [9].

Maintaining good oral function can then be one of the keys to increasing longevity, but the evidence is limited and has not been studied in detail [8]. Thus, additional high-quality studies are needed, and it is extremely important to increase awareness of oral frailty and provide appropriate literature related to this concept to promote healthy aging in the future [5,10].

Therefore, considering the potential impact of the OFI-8 and the need for further validation worldwide, the purpose of this research is to serve as a reference for future studies related to longevity and focused on oral function by translating and exploring the psychometric validity of OFI-8 in the Portuguese population, named the Portuguese version of the OFI-8 (OFI-8-PT).

## Methods

### Design, Setting, and Participants

This was a cross-sectional study that aimed to translate and validate the OFI-8-PT in a sample of patients aged 60 years older from the Egas Moniz Dental Clinic (Almada, Portugal).

To be eligible, participants needed to be aged 60 years or older; be fluent in Portuguese; and consent to participate. The recruitment was carried out between December 30, 2022, and February 17, 2023, which corresponds to a total period of approximately 7 weeks. The recruitment procedure and sampling method consisted of a consecutive convenience sample of participants eligible for participation that presented at the Egas Moniz Dental Clinic for a first dental appointment.

### Sample Size Calculation

To achieve the proposed aim, we calculated a minimum sample size based on the rules of thumb from Terwee et al [14] (between 4 and 10 participants per variable), with a minimum of 10 individuals per questionnaire item. As such, considering an 8-item questionnaire with a dichotomous nature, we estimated a minimum number of 80 participants to ensure the stability of the variance-covariance matrix when performing confirmatory factor analysis (CFA).

### Translation and Cross-Cultural Adaptation of the OFI-8

The original OFI-8 questionnaire measures the oral frailty using 8 dichotomous items, representing the number of natural teeth, masticatory performance, maximum tongue pressure, articulatory oral motor skill, subjective difficulties in eating tough food, and subjective swallowing difficulties.

Each question is assigned a score that is then added together to obtain the total score of the questionnaire, resulting in a numerical scale that ranges from 0 to 11 points; higher scores indicate a greater degree of oral frailty, which in turn has other implications previously described, such as physical frailty, dependence, and mortality [5]. For questions 1, 2, and 3, a response of “Yes” was allocated 2 points, while for questions 4 and 5, a response of “Yes” was allocated 1 point. In contrast, a “No” response was awarded 0 points. Conversely, for questions 6, 7, and 8, a response of “No” was given 1 point, while a response of “Yes” was not awarded any points [10].

Two independent, bilingual individuals fluent in both Portuguese and English translated the original English version of the OFI-8 into Portuguese (VM and JB) in a “double-blind” process. Because no disagreements were recorded, a final version of the questionnaire was named OFI-8-PT. After back-translation to English and comparing with the original to identify discrepancies, no ambiguous wording or cultural nuances were identified. The semantic and conceptual equivalence was attested (Table 1).

**Table 1:** Original version and Portuguese translation of the Oral Frailty Index-8 (OFI-8).

Item	Original (English)	Translation (Portuguese)
1	Do you have any difficulties eating tough foods compared to 6 months ago?	Em comparação com há 6 meses, sentiu dificuldades em comer alimentos duros?
2	Have you choked on your tea or soup recently?	Recentemente, engasgou-se com chá ou sopa?
3	Do you use dentures?	Usa prótese dentária?
4	Do you often have a dry mouth?	Costuma ter a boca seca?
5	Do you go out less frequently than you did last year?	Sai de casa com menos frequência do que no ano passado?
6	Can you eat hard foods like squid jerky or pickled radish?	Consegue comer alimentos duros como carne seca ou nozes?
7	How many times do you brush your teeth in a day? (2 or more times/day)	Quantas vezes escova os dentes por dia (2 ou mais vezes/dia)
8	Do you visit a dental clinic at least annually?	Visita o Médico Dentista pelo menos uma vez por ano?

### Variables

The dependent variable in this study was the older adults’ self-perception of their own oral health (oral frailty score), which occurs as a result of the independent variable. The way in which participants define their oral health condition is used as an indicator for the oral frailty score.

Some parameters of the participants in this study, such as age and sex, were considered independent variables of the study. These variables can be used to determine if there is any relationship with the oral frailty score of the individuals.

### Statistical Analyses

The statistical analysis was conducted in the R *plyr* package (R Foundation for Statistical Computing).

#### Reliability

The OFI-8-PT was pilot-tested on a random sample of 10% of the total sample required for validation (and this subsample was not part of the validation stage). The eligibility criteria of this pilot test were the same as the validation. We used the Kuder-Richardson formula 20 to test the internal consistency of each item, considering its dichotomous nature [15].

#### Validity

Using the *lavaan* package, we performed the CFA to compute the factorial loads and model fitness. To compute the model, we used the maximum likelihood method, with the differences between models being explored through chi-square (*χ*^2^) and likelihood ratio tests. To test the fitness of CFA, we used the *χ*^2^/*df* ratio (good adjustment with values <2) [16], the root mean squared error of approximation (RMSEA; a good model adjustment considered for values between 0.05% and 0.10%; 90% CI) [17], the confirmatory fit index (CFI; a cutoff criterion of ≥0.90 indicates a good fit) [18], goodness-of-fit index (GFI; values of 0.90 or greater indicate well-fitting models) [19], and normed fit index (a cutoff criterion of 0.90) [20].

Then, to make sure that the change of the state variables indicates state variability and not trait change, such as sex, we analyzed the sex invariance of the OFI-8-PT. To do this, we estimated 4 consecutive models: (1) unconstrained model; (2) factor loading–constrained model (Model 1); (3) factor loading– and structural covariance–constrained model (Model 2); and (4) factor loading–, structural covariance–, and measurement residual–constrained model (Model 3). Then, we were able to obtain the Δ values for CFI (ΔCFI) and chi-square (Δ*χ*^2^). We defined a cutoff point of ΔCFI<0.01 as the presence of invariance [18,21] and Δ*χ*^2^=0.095 as invariance between the models [22,23]. In addition, we explored the relationships between the OFI-8-PT items to confirm instrument dimensionality via the Spearman rank correlation coefficient (ρ) and the effect of the previously mentioned 2 confounding variables, sex and age, through the Spearman correlation with the overall OFI-8-PT score. The level of statistical significance was set at 5% in all inferential analyses.

### Ethical Considerations

The data collection was performed electronically, ensuring the confidentiality and anonymity of the participants’ data through a cloud-based system [24]. The study was conducted in accordance with the regulations applicable to research and was approved by the Ethics Committee of the Egas Moniz (ID: 1140/2022). Informed consent was obtained from each participant before their inclusion in the study. Participants entered into this research voluntarily, fully aware of their right to withdraw from the study at any point without the need for justification. The informed consent process ensured that participants had a clear understanding of the study’s purpose, procedures, and the voluntary nature of their involvement. Patients received compensation in the form of free diagnosis and treatment without costs.

## Results

### Participant Characteristics

A total of 159 participants completed the OFI-8-PT, with an average age of 73.9 (SD 9.4; range 60-99) years (mean 74.8, SD 9.7 years and mean 73.1, SD 9.0 years for female and male participants, respectively), and the group was equally composed by female and male participants (n=80, 50.3% female vs n=79, 49.7% male).

### Test-Retest Reliability

To test the reliability of the OFI-8-PT, 16 participants answered the translated tool 2 times (baseline and 2 weeks after). Of these participants, 6 (38%) were female and 10 (62%) were male, with similar age intervals (female: mean 70.8, SD 4.7 years vs male: mean 71.7, SD 9.2 years). The median total score of the instrument was 3 (range 0-7).

The overall internal consistency through Kuder-Richardson formula 20 was 0.69, thus considered to be a good reliability score. Nominally, each question had excellent reliability (Multimedia Appendix 1).

The OFI-8-PT exhibited an adequate reliability (with a Cronbach α coefficient of 0.95) and an adequate psychometric feature.

### Construct Validity

The CFA analysis attested the unifactorial structure of the OFI-8-PT. The first-order unifactorial model resulted in an adequate model fit (GFI=0.96; CFI=0.85; and RMSEA=0.05, 90% CI 0.00-0.09; Table 2).

**Table 2:** Model fit indices in the unifactorial model and configurational invariance by sex.

Model			*χ* ^2^		*df*		*P* value		*χ*^2^/*df*		CFI^a^		GFI^b^		RMSEA^c^ (90% CI)		ΔCFI		Δ*χ*^2^		Δ*df*
**Unifactorial**			26.5		20		<.001		1.33		0.85		0.96		0.05 (0.00-0.09)		—^d^		—		—
**Measurement invariance across sex**																					
	Unconstrained	43.8		40		—		1.10		0.90		0.96		0.04 (0.00-0.09)		—		—		—	
	1	48.6		47		<.001		1.03		1.00		0.96		0.00 (0.00-0.07)		0.10		4.8		7	
	2	56.0		54		<.001		1.04		0.95		0.99		0.02 (0.00-0.08)		0.05		7.4		7	
	3	56.0		54		<.001		1.04		0.95		0.99		0.02 (0.00-0.08)		0.00		0.0		0	

^a^CFI: comparative fit index.

^b^GFI: goodness-of-fit index.

^c^RMSEA: root mean square error of approximation.

^d^Not applicable.

A multigroup CFA assessed the invariance across sex groups in the OFI-8-PT (Table 1). Accordingly, there was invariance for sex groups based on the comparisons of CFI, *χ*^2^, and *df* values across the unconstrained and constrained models studied.

In order to learn the agreement level of OFI-8-PT components, we analyzed the correlation between the items using Spearman rank correlation coefficient. There was a low number of significant correlations (4/28, 14% of all correlations; Table 3).

**Table 3:** Correlation between Portuguese version of the Oral Frailty Index-8 item scores.

Item		1	2	3	4	5	6	7	8
**1**									
	*r*	1	0.103	0.166	–0.04	0.215	0.331	0.112	0.066
	*P* value	—^a^	>.99	.38	.61	.29	.01	.49	.49
**2**									
	*r*	0.103	1	0.175	0.044	0.075	0.07	0.012	0.133
	*P* value	>.99	—	.21	.91	.29	.49	.16	.74
**3**									
	*r*	0.166	0.175	1	0.117	0.024	0.205	–0.105	–0.037
	*P* value	.38	.21	—	.14	.09	.23	.09	.047
**4**									
	*r*	–0.04	0.044	0.117	1	0.086	0.102	–0.042	–0.005
	*P* value	.61	.91	.14	—	.14	.91	.12	.16
**5**									
	*r*	0.215	0.075	0.024	0.086	1	0.142	0.187	0.145
	*P* value	.29	.29	.09	.14	—	.78	.03	.18
**6**									
	*r*	0.331	0.07	0.205	0.102	0.142	1	0.181	–0.009
	*P* value	.01	.49	.23	.91	.78	—	.78	.047
**7**									
	*r*	0.112	0.012	–0.105	–0.042	0.187	0.181	1	0.123
	*P* value	.49	.16	.09	.12	.03	.78	—	.23
**8**									
	*r*	0.066	0.133	–0.037	–0.005	0.145	–0.009	0.123	1
	*P* value	.49	.74	.047	.16	.18	.047	.23	—

^a^Not applicable.

### Confounding Variables

When correlating the overall OFI-8-PT score, age was significantly correlated (ρ=0.259; *P*<.001), whereas sex was not (ρ=–0.035; *P*=.66), confirming as well the absence of sex invariance.

## Discussion

### Principal Findings

This study demonstrated the psychometric validity of the OFI-8-PT to depict individuals at risk of oral frailty and functional disability in this Portuguese sample. In addition, we found an adequate internal consistency and reliability of this questionnaire.

The assessment of oral frailty in older adult individuals through a comprehensive scale holds great clinical significance due to its potential influence on geriatric care and the overall health outcomes of these individuals. Older adults typically face a variety of oral health challenges, such as tooth loss, periodontal disease, and dry mouth, which can significantly impact their quality of life and general well-being [25,26]. Despite the prevalence of oral health problems in the older adult population, there is a scarcity of specialized tools designed to assess oral frailty, which encompasses the functional and structural vulnerabilities of the oral cavity [27,28].

Expanding the usability of the OFI-8 to other languages could help health care professionals detect, intervene, and track oral health in older adults, potentially improving their quality of life through preventive care and tailored hygiene. Moreover, incorporating oral frailty assessment into routine geriatric assessments can facilitate comprehensive care planning and interdisciplinary collaboration among health care providers [29]. By addressing the clinical need for a validated scale to assess oral frailty in older adults, this study contributes to enhancing the holistic approach to geriatric health care and underscores the importance of oral health in promoting healthy aging.

The GFI indicated that the observed covariance matrix of the data fitted the covariance matrix implied by the model, revealing a very good fit. The CFI, however, suggested a reasonable fit with room for improvement when compared to a null model. The RMSEA also revealed a good fit with a value of 0.05. These results support the validity of the OFI-8-PT, although they cannot be directly compared with the original study by Tanaka et al [5] due to differences in methodology. Additionally, this is the first study to validate the OFI-8 in a language other than Japanese, which may contribute to future comparability with other validations and cultural adaptations.

With the introduction of the OFI-8-PT, health professionals and researchers will have a valuable tool for assessing oral frailty in older adults in Portugal. This standardized and validated questionnaire will enable early detection of oral frailty, allowing for targeted interventions and preventive strategies. With the current aging population, understanding and managing oral frailty is becoming increasingly important to promote healthy aging and improve overall well-being. By identifying individuals at risk, the OFI-8-PT can facilitate appropriate oral health services, ultimately contributing to improved oral health outcomes and quality of life.

The OFI-8-PT questionnaire offers several advantages that make it a valuable tool for assessing oral frailty in older adults in Portugal. Its standardized and validated nature ensures reliable and consistent assessment, while its ease of administration and scoring facilitates its incorporation into routine assessments. Implementation of the OFI-8-PT also allows for data collection on oral frailty in the Portuguese population, contributing to a better understanding of its prevalence and impact on health outcomes. However, it is important to acknowledge potential drawbacks, such as its primary focus on oral health aspects and its reliance on self-reporting, which may introduce bias or inaccuracy. Addressing these limitations and ensuring proper training and awareness of participants will be critical to maximizing the benefits of the OFI-8-PT questionnaire.

### Strengths and Limitations

Overall, the study provides valuable insights into the psychometric validity of the OFI-8-PT questionnaire in the Portuguese population. However, there are some important limitations worth discussing that should be considered when interpreting the results.

First, the sample size used in the study was relatively small, which may limit the generalizability of the findings to a larger population. Yet, this shortcoming is limited as the OFI-8 is a 1-factor scale and we followed a previous validated strategy. Additionally, the study relied solely on self-report measures, based on self-perception of oral health, which could result in bias or social desirability effects. Furthermore, the study was conducted in a specific population from a university clinic, consisting solely of patients who visited the dental clinic, and therefore, findings may not be generalizable to the entire Portuguese population or other populations in different geographical locations. Nevertheless, these participants live in the largest metropolitan area in Portugal and may provide a considerable percentage of representation. Moreover, the presence of confounding variables, such as chronic diseases and medications, could influence the oral frailty score of individuals. Although these variables were acknowledged, the study did not extensively analyze their impact or control for their effects, and the study could not assess longitudinal effects due to its design.

Accordingly, conducting further research with larger and more diverse samples is recommended to address these limitations and strengthen the validity of the findings.

### Conclusion

In conclusion, the OFI-8-PT was shown to have psychometric validity and reliability in the population under study, providing a snapshot of its oral frailty and functional disability. This consistency and ease of use may contribute to better screening and monitoring oral frailty prevalence and its impact on health outcomes. It may have an impact on future public health strategies to address oral frailty.

## Appendix

Multimedia Appendix 1 Reliability for each question of the Portuguese version of the Oral Frailty Index-8.

## Abbreviations

CFA: confirmatory factor analysis
CFI: comparative fit index
GFI: goodness-of-fit index
OFI-8: Oral Frailty Index-8
OFI-8-PT: Portuguese version of the Oral Frailty Index-8
RMSEA: root mean square error of approximation

## Data Availability

The datasets generated during and analyzed during this study are available in a Zenodo repository [30].
