# Supplementary material for: Portuguese Version of the Oral Frailty Index-8: Instrument Validation Study
Source: Interact J Med Res. 2024 Oct 28;13:e49975. doi: 10.2196/49975 (PMC11555463; doi:10.2196/49975)
Supplement: Multimedia Appendix 1 [file ijmr_v13i1e49975_app1.docx]

**Supplementary Files**

Table S1. Original version and Portuguese translation of the OFI-8.

|  | Original (EN) | Portuguese (PT) |
| --- | --- | --- |
| Item 1 | Do you have any difficulties eating tough foods compared to 6 months ago? | Em comparação com há 6 meses, sentiu dificuldades em comer alimentos duros? |
| Item 2 | Have you choked on your tea or soup recently? | Recentemente, engasgou-se com chá ou sopa? |
| Item 3 | Do you use dentures? | Usa prótese dentária? |
| Item 4 | Do you often have a dry mouth? | Costuma ter a boca seca? |
| Item 5 | Do you go out less frequently than you did last year? | Sai de casa com menos frequência do que no ano passado? |
| Item 6 | Can you eat hard foods like squid jerky or pickled radish? | Consegue comer alimentos duros como carne seca ou nozes? |
| Item 7 | How many times do you brush your teeth in a day? (2 or more times/day) | Quantas vezes escova os dentes por dia (2 ou mais vezes/dia) |
| Item 8 | Do you visit a dental clinic at least annually? | Visita o Médico Dentista pelo menos uma vez por ano? |

Table S2. Test-retest reliability using Cronbach’s alpha for OFI-8

|  | Cronbach’s α Coefficient (95% CI) |
| --- | --- |
| Item 1 | 0.99 (0.97-1.00) |
| Item 2 | 0.97 (0.93-0.99) |
| Item 3 | 0.94 (0.88-0.97) |
| Item 4 | 0.93 (0.88-0.97) |
| Item 5 | 0.91 (0.83-0.95) |
| Item 6 | 0.90 (0.84-0.95) |
| Item 7 | 0.77 (0.55-0.91) |
| Item 8 | 0.79 (0.54-0.95) |
| OFI-8 Total | 0.95 (0.81-0.99) |

CI—confidence interval; ICC—intraclass correlation coefficient.
